# Supplementary material for: Extinction Risk Assessment and Chemical Composition of Aerial Parts Essential Oils from Two Endangered Endemic Malagasy Salvia Species
Source: Plants (Basel). 2023 May 12;12(10):1967. doi: 10.3390/plants12101967 (PMC10223310; doi:10.3390/plants12101967)
Supplement: Supplementary file 1 [file plants-12-01967-s001.zip › plants-2328787-supplementary.pdf]

# Extinction Risk Assessment and Chemical Composition of Aerial Parts Essential Oils from Two Endangered Endemic Malagasy *Salvia* Species

**Stéphan R. Rakotonandrasana[1], Mathieu Paoli[2], Mamy J. Randrianirina[3], Harilala Ihandriharison[3], Marc Gibernau[2], Ange Bighelli[2]; Marrino F. Rakotoarisoa[1], Pierre Tomi[2], Charles Andrianjara[3], Félix Tomi[2,*] and Delphin J. R. Rabehaja[3]**

[1] Department of Ethnobotany and Botany, National Center for Applied Pharmaceutical Research, 101 Antananarivo, Madagascar; stephanandrasana@gmail.com (S.R.R.); rino2r@hotmail.fr (M.F.R)

[2] Laboratoire Sciences pour l'Environnement, Université de Corse-CNRS, UMR 6134 SPE, Route des Sanguinaires, 20000 Ajaccio, France; paoli_m@univ-corse.fr (M.P.); gibernau_m@univ-corse.fr (M.G.), bighelli_a@univ-corse.fr (A.B.), tomi_p@univ-corse.fr (P.T.) ; tomi_f@univ-corse.fr (F.T.)

[3] Institut Malgache de Recherches Appliquées (IMRA), Département Phytochimie et Contrôle Qualité, Antananarivo 102, Madagascar ; rabehaja@yahoo.fr (D.J.R.R.); charles.andrianjara@gmail.com (C.A.); mjrandrianirina@gmail.com (M.J.R.); iharilalandr@gmail.com (H.I.)

* Correspondence: tomi_f@univ-corse.fr; Tel.: +33-495-52-4122

**Table S1.** Main components of Salvia Essential Oils from East Africa

| Country | **aethopia** [15, 16] | | | **somalia** [17] | | **South Africa** [18-20] | | | | | | | | | | |
|---|---|---|---|---|---|---|---|---|---|---|---|---|---|---|---|---|
| **Compounds** **Species** | *Sae* | *Sni* | *Ssc* | *Sdo* | *Sso* | *Sac* | *Sal* | *Sab* | *Sch* | *Sdo* | *Sla* | *Smu* | *Sra* | *Sre* | *Sru* | *Sst* |
| α-Pinene | | | | 9.6 | 9.3 | 4.1 | 6.0 | | 10.4 | | 2.6 | 22.3 | | | | |
| Camphene | | | | 5.2 | 8.0 | | | | | | | 7.6 | | 3.3 | | 2.0 |
| β-Pinene | | | | | | | | | | | | 7.6 | | | | |
| Myrcene | | | | 2.7 | | | 11.5 | | | | | | | | | 3.9 |
| δ-3-Carene | | | | 6.7 | 6.3 | | | | | | | | | | | 22.6 |
| *p*-Cymene | | | | | | | 7.6 | 2.5 | | | | | | | | |
| Limonene | | | | 8.9 | 4.6 | | | 9.4 | 9.7 | | | 11.6 | | 9.4 | | 4.8 |
| β-Phellandrene | | | | | | | | | | | | | | | | 3.7 |
| 1,8-Cineole | | | | 18.9 | | | | 9.4 | 40.5 | | | 23.2 | | 12.8 | | |
| (*Z*)-β-Ocimene | | | | 2.7 | | 2.6 | 5.4 | | | | | | | | | |
| Linalool | | | 44.4 | | | | | | | 16.6 | | | | | | |
| Camphor | | | | | 12.5 | | 5.4 | | | | | 8.3 | | 5.3 | | 3.2 |
| Borneol | | | | 6.6 | 2.8 | | | | | | | | | | | 2.2 |
| Terpinen-4-ol | | | | | | | | | | | | | | | | |
| α-Terpineol | | | 9.3 | | | | | 2.7 | | 6.2 | | | | | | |
| Geraniol | | | | | | | | | | 19.6 | | | | | | |
| Linalyl acetate | | | | | | | | | | 19.6 | | | | | | |
| Bornyl acetate | | | | | 16.2 | | | | | | | | | | | |
| α-Copaene | 19.8 | | | | 3.5 | | | | | | | | | | | |

| | | | | | | | | | | | | | | | | |
|---|---|---|---|---|---|---|---|---|---|---|---|---|---|---|---|---|
| β-Cubebene+ β-Elemene | 9.9 | | | | | | | | | | | | | | | |
| (*E*)-β-Caryophyllene | 7.3 | 13.0 | | 13.1 | 4.5 | | 3.4 | 2.1 | | | 5.7 | 2.2 | | 8.0 | 10.5 | 2.1 |
| α-Humulene | | 3.8 | | | | | | | | | 4.7 | | | 2.8 | 2.3 | |
| Germacrene D | 29.0 | 28.5 | | | | | | | | | | | | | | |
| Bicyclogermacrene | 9.3 | 8.4 | | | | | | | | | | | | | | |
| δ-Cadinene | 8.7 | | | 2.5 | 6.4 | | | | | | | | | | | |
| (E)-Nerolidol | | | | | | | 3.5 | 2.1 | | | 2.2 | | | | | |
| Caryophyllene oxide | | | | | | 14.6 | | 5.6 | | | 14.3 | | 22.6 | 4.9 | 1.3 | |
| Spathulenol | | | | | | 29.1 | 2.0 | 2.0 | | | 18.3 | | 7.7 | 2.6 | | |
| Humulene oxide I | | | | | | | | | | | | | 12.6 | | | |
| Viridiflorol | | | | | | | 3.3 | 24.5 | 9.3 | | | | | 5.3 | | |
| Ledol | | | | | | 6.5 | | 6.6 | | | 5.2 | | | 4.6 | | |
| Guaiol | | 14.0 | | | | | | | | | | | | | | |
| τ-Cadinol | | | | | | 3.0 | | 2.0 | 2.3 | | | | | | | |
| α-Bisabolol | | | | | | | | | | | | | 3.8 | | 65.5 | 26.1 |

Percentage of reported components ≥ 5%

*Sae = Salvia aethiopis; Sni = S. nilotica; Ssc = S. schimperi; Sdo = S. dolomitica; Sso = S. somaliensis; Sac = S. africana-caerulea; Sal = S. africana-lutea; Sab = S. albicaulis; S. chamelaegnea; Sdo = S. dolomitica; Sla = S. lanceolate; Smu = S. muirii; Sra = S. radula; Sre = S. repens; Sru = S. runcinata; Sst = S. stenophylla.*

## References


15. Güllüce, M.; Özer, H.; Bariş, Ö.; Dafedera, D.; Şahin, F.; Polissiou, M. Chemical composition of the essential oil of *S. aethiopis* L. *Turk J. Biol*. 2**006**, *30(4)*, 231-235.
16. Ashafa, H.; Asres, K.; Mazumder, A.; Bucar A. Leaf essential oil of *S. nilotica* and *S. schimperi*: their antimicrobial and antioxidant activities. *Ethiop. Pharm. J*. **2008,** *26*, 49-58.
17. Ebani, V.V.; Nardoni, S.; Bertelloni, F.; Giovanelli, S.; Ruffoni, B.; D'Ascenzi, C.; Pistelli, L.; Mancianti, F. Activity of *S. dolomitica* and *S. somaliensis* essential oils against bacteria, molds and yeasts. *Molecules*. **2018**, *3*, 396.
18. Kamatou, G.P.P.; Van Zyl, R. L.; Van Vuuren, S.F.; Viljoen, A.M.; Figueiredo, A.C.; Barroso, J.G., Pedro, L.G. Biological activities and composition of *Salvia muirii* L. Bol. Essential oil. *J. Essent. Oil Res.* **2006,** *18*, 48-51.
19. Kamatou, G.P.P.; Van Zyl, R. L.; Van Vuuren, S.F.; Viljoen, A.M.; Figueiredo, A.C.; Barroso, J.G., Pedro, L.G.; Tilney P.M. *J. Essent. Oil Res*. **2006**, *18*, 72-79.
20. Kamatou, G.P.P. Indigenous *Salvia* species – An investigation of their pharmacological activities and phytochemistry. Thesis, Doctor of Philosophy, University of the Witwatersrand, Johannesburg, 2006.
